# Restriction of YWHAB-mediated YAP cytoplasmic retention is a novel mechanism underlying stemness maintenance and chemoresistance in ovarian cancer peritoneal metastasis

**DOI:** 10.1016/j.gendis.2025.101519

**Published:** 2025-01-08

**Authors:** Chang Liu, Lei Shi, Zijun Meng, Manlin Zhang, Zhiqi Zhang, Yunzhe Li, Kaiwen Du, Muyao Yang, Lin Qiu, Jing Feng, Yuchen He, Jiayun Liu, Hua Zhang, Hongbin Zhang, Tingyuan Lang, Zhuo Yang

**Affiliations:** a Department of Gynecology, Cancer Hospital of Dalian University of Technology, Cancer Hospital of China Medical University, Liaoning Cancer Hospital & Institute, Shenyang, Liaoning 110001, China; b Hematology and Oncology Department, The People's Hospital of Tongliang District, Chongqing 402560, China; c Reproductive Medicine Center, The First Affiliated Hospital of Chongqing Medical University, Chongqing 400016, China; d Chongqing Key Laboratory of Translational Medical Research in Cognitive Development and Learning and Memory Disorders, Ministry of Education Key Laboratory of Child Development and Disorders, National Clinical Research Center for Child Health and Disorders, China International Science and Technology Cooperation Base of Child Development and Critical Disorders, Children's Hospital of Chongqing Medical University, Chongqing 400015, China; e Department of Obstetrics and Gynecology, Shengjing Hospital of China Medical University, Shenyang, Liaoning 110136, China; f Department of General Surgery, Shanghai Fourth People's Hospital, School of Medicine, Tongji University, Shanghai 200434, China; g Department of Gynecology and Obstetrics, The First Affiliated Hospital of Chongqing Medical University, Chongqing 400016, China; h Department of Gynecology, The First Branch of The First Affiliated Hospital of Chongqing Medical University, Chongqing 400042, China; i State Key Laboratory of Maternal and Fetal Medicine of Chongqing, Chongqing Medical University, Chongqing 400016, China; j College of Bioengineering, Chongqing University, Chongqing 400044, China; k Department of Clinical Laboratory, Xijing Hospital, Fourth Military Medical University, Xi'an, Shannxi 710032, China; l Department of Hematology, The First Affiliated Hospital of Chongqing Medical University, Chongqing 400016, China

**Keywords:** Chemoresistance, Ovarian cancer, Peritoneal metastasis, Stemness, YAP, YWHAB

## Abstract

Ovarian cancer (OC) peritoneal metastasis (OCPM) is a major cause of high mortality of OC, in which cancer cells incubated in ascites evolve various mechanisms to survive. Hippo/YAP singling plays multiple roles in carcinogenesis, however, its roles in OCPM have remained elusive. Here, we report that restriction of YWHAB-mediated YAP cytoplasmic retention is a critical mechanism underlying OCPM stemness maintenance. Combined tandem mass tag- and tissue microarray-based proteomic studies revealed YWHAB down-regulation in post-neoadjuvant chemotherapy OCPM tissues, which was confirmed in no-neoadjuvant-chemotherapy-response tissues, isolated OCPM stem cells, and induced cisplatin-resistant cells. Knockdown of YWHAB promoted stemness and resistance in parental complete or near-complete primary OCPM and OVCAR3 cells *in vitro* and *in vivo*. Mechanistic study showed that YWHAB directly bound to YAP and promoted YAP cytoplasmic retention and thus YWHAB restriction promoted YAP activity and stemness in OCPM in the cells in which the Hippo/YAP signaling was constitutively activated by overloaded constitutively active YAP (YAP5SA), and the effect of YWHAB knockdown was significantly abolished. The SH3 binding domain in YAP is critical for YWHAB-YAP binding. Alteration in the 5mc methylation level in the YWHAB promoter was observed in OCPM stem cells. In summary, our results reveal that restriction of YWHAB-mediated YAP cytoplasmic retention is a critical mechanism underlying OCPM stemness maintenance. Our findings suggest that YAP would be a therapeutic target for suppressing OCPM stemness caused by YWHAB restriction.

## Introduction

Ovarian cancer (OC) is the leading cause of death in gynecologic cancers.^1^ OC peritoneal metastasis (OCPM) is the hallmark event, contributing to the high mortality, in which the cancer cells sporadically grow on the inner surface of the parietal peritoneum or visceral peritoneum and evolute various mechanisms in the ascites microenvironment for survival; unfortunately, around 75% of patients had OCPM when first diagnosed as OC.^2^ The current primary treatment for this advanced OC is neoadjuvant chemotherapy (NACT, platinum-based drugs with taxane) followed by interval debulking surgery; however, even if a good response and R0 resection were achieved, the cryptic residual cancer cells would develop chemoresistance, finally leading to distant metastasis.^3^^,^^4^ Thus, developing strategies for reversing OCPM chemoresistance is urgently needed.

Evidence supports that cancer stem cells, a subpopulation of tumor cells with stem-like properties, are enriched in residual tumors, which are primarily responsible for recurrence, metastasis, and therapeutic resistance.^5^ Especially in OCPM, the ascites microenvironments in the abdominal cavity are suitable for ovarian cancer stem cell transformation.^6^^,^^7^ Therefore, targeting OCPM stem cells (OCPM-SCs) would be an effective therapeutic strategy. However, rare studies have been performed to investigate OCPM stemness.

Hippo (also known as Hippo/YAP) signaling is a kinase cascade composed of MST1/2 and LATS1/2 kinases. MST1/2 phosphorylate and activate LATS1/2, which in turn phosphorylate the Yes-associated protein (YAP) coactivator.^8^ YAP is a transcription coactivator that acts via forming a complex with transcriptional enhanced associate domain (TEAD) transcription factor.^9^ Phosphorylation of YAP recruits YWHA family proteins and leads to cytoplasmic retention of YAP.^10^ The phosphorylation of YAP is also regulated by other kinases or phosphatases, such as protein phosphatase 2A (PP2A).^8^^,^^9^ Hippo signaling plays important roles in organ development and cancer, however, the roles of Hippo signaling in OCPM remain elusive.

In this study, we first found that YWHAB expression was suppressed in post-NACT and no-response OCPM tissues, isolated OCPM-SCs, and induced resistant OCPM cells, followed by a demonstration of YWHAB as a negative regulator for OCPM stemness via *in vitro* and *in vivo* experiments. Mechanistic studies reveal that restriction of YWHAB-mediated YAP cytoplasmic retention is the underlying mechanism. These findings thus reveal a novel mechanism involved in OCPM stemness and suggest the potential application of YAP suppression in OCPM treatment.

## Materials and methods

### Clinical samples and ethical issues

The fresh OCPM tumor samples were collected from patients with high-grade serous ovarian cancer during laparoscopic or debulking surgery at the Cancer Hospital of Dalian University of Technology (Dalian, China). All patients were classified into no or minimal response (NR), partial response (PR), and complete or near-complete (CR) response to NACT. In this study, PR and CR are pathological responses based on a standardized chemotherapy response scoring system developed by Böhm et al^11^ in 2015, while NR is a radiological response assessed by computed tomography imaging according to Response Evaluation Criteria In Solid Tumors (RECIST).^12^ Three paired pre- and post-NACT OCPM tissues from 3 patients were collected for proteomic analysis. In total, 53 pairs of pre- and post-NACT PR OCPM tissues as well as 14 CR and 100 NR pre-NACT OCPM tissues were collected for further immunohistochemistry analysis. The primary cells isolated from the OCPM tissues were developed. The clinical information of the samples is provided in Table S1.

### Comparative proteomic study

Both the tandem mass tag- and tissue microarray-based proteomic studies were employed in this study. A tandem mass tag-based proteomic study was performed according to the previously reported protocol.^13^ Briefly, the proteins were extracted according to the protocols reported by Zhang et al.^14^ The samples were minced into small species. The lysis was performed in a lysis buffer (8 M urea, 100 mM Tris hydrochloride, pH 8.0) supplemented with protease and phosphatase inhibitors. Sonication was subsequently performed. After centrifugation, the supernatant (protein extract) was collected, followed by reduction (10 mM dithiothreitol at 56 °C for 60 min) and alkylation (10 mM iodoacetamide at room temperature in the dark for 60 min). The proteins were then digested into peptides with trypsin. Finally, a C18 column was used for desalination. The desalinated peptides were resolved in 100 mM triethylamine buffer. TMT® Mass Tagging Kits and Reagents (Thermo Scientific) were used for labeling. Mass spectrometry detection was performed on a Q Exactive HF-X Hybrid Quadrupole-Orbitrap Mass Spectrometer (ThermoFisher Scientific) coupled with an EASY nLC 1200 high-performance liquid chromatography system (ThermoFisher Scientific). Mass spectrometry raw files were searched against the NCBI human Refseq protein database by Proteome Discoverer (version 2.4.1.15, ThermoFisher Scientific). The proteins containing at least one unique peptide with FDR < 1% were identified. The total peptide amount was used for normalization. Default setup was used for other parameters. Features with more than 30% missing values were excluded. Outliers were identified by mean ± three standard deviations. The missing values and outliers were imputed by the KNN algorithm. The differentially expressed proteins were defined as *p* < 0.05 and fold change >1.5. For tissue microarray-based experiments, tissue microarrays were constructed using 53 pairs of pre- and post-NACT PR OCPM tissues according to the protocol previously described.^15^ Tissue microarray slides were deparaffinized and rehydrated, followed by antigen retrieval. After blocking, primary antibodies and a second HRP-conjugated antibody were applied. The following antibodies were used: phospho-MST1 (STK4) (Thr183) antibody (PA5-40177, ThermoFisher Scientific, 1:500), phospho-MST2 (Ser316) antibody (PA5-105631, ThermoFisher Scientific, 1:600), phospho-LATS1 (Ser909) antibody (#9157, Cell Signaling Technology, 1:500), phospho-LATS2 (Ser872) antibody (#AF7440, Affinity Biosciences, 1:500), YAP (#14074, Cell Signaling Technology, 1:800), 14-3-3 gamma/YWHAG antibody (ab155050, Abcam, 1:600), 14-3-3 tau/YWHAQ antibody (ab264319, Abcam, 1:500), 14-3-3 beta/alpha/YWHAB (#PA1-37002, ThermoFisher Scientific, 1:600), 14-3-3 Epsilon/YWHAE antibody (ab92311, Abcam, 1:500), PPP2CA antibody (ab106262, Abcam, 1:800), and PPP2CB antibody (ab168371, Abcam, 1:500).

### Bioinformatic analysis

Pathway, cellular function, and gene ontology (GO) analysis were performed using the online tool REACTOME.^16^

### Cell culture

The OVCAR3 (epithelial cells isolated from the malignant ascites of a patient with progressive ovarian cancer) cell line was purchased from The American Type Culture Collection. The OVCAR3 cells were cultured in RPMI-1640 medium supplemented with 0.01 mg/mL bovine insulin and 20% fetal bovine serum. Short tandem repeat profiling was adopted to validate the cell line. The primary cells were isolated and cultured according to previously reported protocol.^17^ Briefly, solid OCPM specimens were placed in a sterile, screw lid, polypropylene container filled with ice-cold phosphate-buffered saline. After being transferred to the laboratory, the specimens were transferred onto a Petri dish containing fresh, ice-cold phosphate-buffered saline. The specimens were further cut into pieces (about 2 mm in size) for optimal exposure of the specimens to enzyme (prewarmed dispase II (2.4 U/mL) at 24 °C for 30 min). After digestion, a cell strainer (70 uM mesh) was used to make a single-cell suspension. The cells were collected by centrifugation and resuspended in 10 mL of DMEM medium containing 10% fetal bovine serum and incubated at 37 °C and 5% CO_2_. The medium was changed after 24 h from the initial plating to remove the cellular debris. Then, the medium was changed every 72 h. The primary cells usually exhibited typical epithelial cobblestone morphology after two weeks in culture. As fibroblasts usually detach faster than tumor cells, the fibroblasts can be removed according to the following protocols. The cells were incubated with trypsin/EDTA at 37 °C for 0.5–1 min intervals. After each interval, the supernatant was aspirated. After 5–10 cycles, the tumor cells still attached to the plate were washed and cultured. The primary cells usually can be passaged 25 times and the primary cells within passages 3–8 were used in routine experiments, except in experiments involved in induced resistant cells. Mycoplasma contamination was detected by a polymerase chain reaction-based mycoplasma detection kit (#4460626, ThermoFisher Scientific) for all cells.

### Immunohistochemistry assay

Formalin-fixed and paraffin-embedded samples were used in the study. Deparaffinization and rehydration were performed by incubation with xylene and ethanol. Antigen unmasking was performed by boiling the slides in 1 mM EDTA (pH 8.0). The slide was then incubated in 3% hydrogen peroxide for 10 min, followed by incubation in a blocking buffer at 25 °C for 1 h. The side was then incubated with primary and secondary antibodies successively, followed by hematoxylin staining. The antibody YWHAB (#PA1-37002, ThermoFisher Scientific, 1:1000) was used. The slide was then dehydrated with ethanol and cleared with xylene, followed by mounting with a resin mounting medium. Immunoreactivity score, a combination of staining intensity and proportion of positive cells,^18^ was used to assess the expression level of the target proteins.

### Isolation of OCPM-SCs

OCPM-SCs were isolated and cultured according to previously reported protocol.^16^ Briefly, the adherent parental cells were seeded in ultra-low attachment culture dish/plate (#3471/4520, Corning) containing serum-free medium supplemented with 20 ng mL-1 recombinant human epidermal growth factor (rh-EGF, PHG0313, ThermoFisher Scientific), 10 ng mL-1 recombinant human basic fibroblast growth factor (rh-bFGF, PMG0033, ThermoFisher Scientific), and antibiotics. After the spheroid cells reached 60% confluence, CD133^+^ spheroid cells were separated by magnetic-assisted cell sorting technology (#130-100-857, Miltenyi Biotech) according to the manual. CD133^+^ ALHD^+^ spheroid cells were then isolated from CD133^+^ cells by ALDEFLUOR™ Kit (#01700_C, Stem Cell Technologies) according to the manual, which were named as OCSLCs.

### Generation of cisplatin-resistant OCPM cells

Cisplatin (cis-diammineplatinum (II) dichloride, #22-515-0, ThermoFisher Scientific) was dissolved in 0.15M NaCl and stored at −80 °C. The experiments were conducted according to previously reported protocol.^19^ Cisplatin-resistant variants of primary cells and OVCAR3 cells were derived by continuous exposure of original parental cells to cisplatin (IC50 values). The IC50 values were obtained by dose–response study. In each cycle, the parental cells were treated with cisplatin (IC50) for 72 h, followed by 72-h recovery. This task was repeated until at least 3-fold IC50 was obtained. The resistant cells were then cultured with the complete medium containing cisplatin (IC10) to maintain the resistance. The primary cells within passage 5 were used for the generation of cisplatin-resistant OCPM cells, and the developed induced cisplatin-resistant OCPM cells should be used within passage 15. The IC_50_ values were measured at 72 h after drug treatment.

### Quantitative PCR

Total RNA was extracted using TRIzol reagent (#15596026 Thermofisher Scientific). Quantitative PCR was performed using a one-step probe qPCR mix ROX (#AM09.1, QIAGEN), containing one-step probe qPCR mix, high/low ROX solution, RNase inhibitor, and reverse transcriptase, according to the manufacturer's instructions. Each experiment was performed in triplicate. The information of primers is listed in Table S2.

### ALDH activity assay

ALDH activity was examined by ALDEFLUOR™ Kit (#01700, Stem Cell Technologies) according to the manual. Briefly, the cells were collected and added into ALDEFLUOR™ Assay Buffer at a concentration of 1 × 10^6^ cells/mL. The substrate was then added to the buffer and incubated at 37 °C for 60 min. After washing with the buffer, the fluorescent signal was detected by a plate reader or flow cytometry.

### Protein preparation, Western blot, and co-immunoprecipitation

Whole-cell proteins were extracted by RIPA lysis buffer according to the standard protocol. Nuclear and cytoplasmic proteins were prepared by NE-PER nuclear and cytoplasmic extraction reagents (#78833, ThermoFisher Scientific) according to the manual. Briefly, the samples in the tube were incubated on ice for 10 min. Ice-cold cytoplasmic extraction reagent (CER II) was added to the tube, followed by vortex (5 s on the highest setting) and incubation (1 min) on ice. After another vortex (5 s on the highest setting), the tube was centrifuged (16,000 *g*, 5 min), and the supernatant (cytoplasmic extract) was transferred to a clean pre-chilled tube. The pellet fraction, which contained nuclei, was resuspended by an ice-cold nuclear extraction reagent. The tube was subjected to vortex (5 s on the highest setting) every 10 mi for a total of 40 min. After centrifugation (16,000 *g*, 5 min), the supernatant (nuclear extract) fraction was transferred to a clean pre-chilled tube. Western blot was performed according to standard protocols. The following antibodies were used: YWHAB (#PA1-37002, ThermoFisher Scientific, 1:2000), β-actin (#4970, Cell Signaling Technology, 1:4000), YAP (#14074, Cell Signaling Technology, 1:2500), phosphorylated YAP (#13008, Cell Signaling Technology, 1:2500), Lamin B1 (#13435, Cell Signaling Technology, 1:4000), and Pan-TEAD (#13295, Cell Signaling Technology, 1:4000). Co-immunoprecipitation assays were performed by a co-immunoprecipitation kit (#26149, ThermoFisher Scientific) according to the manual. Briefly, the purified antibodies were covalently coupled onto the amine-reactive resin. The antigens (bait protein) with its complex were linked to the resin, followed by elution. The following antibodies were used to develop antibody-linked beads: Pan-TEAD (#13295, Cell Signaling Technology) and YAP (#14074, Cell Signaling Technology).

### Sphere formation assay

Sphere formation assay was performed according to the previously reported protocol.^20^ Briefly, single-cell suspension was prepared by dissociation and a cell strainer (70 uM mesh). The cells were seeded in a 6-well ultra-low attachment culture plate (#3471, Corning) containing serum-free SCSLC culture medium at the density of 3000 cells per well. The spheroids were examined after 11–21 days of culture. For each biological replicate, the average diameter and number of three independent fields of view were recorded.

### Xenograft

Female BALB/c nude mice (Pusheng Technology, Nanjing, China), 6–8 weeks of age, were employed. The cells were suspended in 0.2 mL culture medium containing 25% Matrigel (#CB-40234, ThermoFisher Scientific) and inoculated into the flank of the mice. The tumor volume (length × width^2^/2) was recorded every 7 days. All animal experiments were approved by the Institutional Animal Care and Use Committee, Cancer Hospital of Dalian University of Technology (Dalian, China).

### Limiting dilution assay

The limiting dilution assay was performed according to previously reported protocols.^17^^,^^20^ Briefly, for *in vitro* assay, the cells were seeded into a 96-well ultra-low attachment culture plate (#3474, Corning) at the density of 1, 5, and 10 cells per well (50 wells for each group). The number of wells containing spheroids was recorded after culture for 15–25 days. For the *in vivo* assay, the cells were inoculated into the mice at a density of 8, 40, and 200 cells per mouse (10 mice for each group). The number of tumor-bearing mice was recorded at the endpoint. An online tool was performed for extreme limiting dilution analyses.^21^

### Fluorescence-activated cell sorting analysis

Fluorescence-activated cell sorting assay was employed to assess the percentage of ovarian cancer stem cells in the cultured cells according to the standard protocol. Briefly, the cultured cells were digested by trypsin and collected by centrifugation. One million cells were incubated with antibodies at 4 °C in the dark for 30 min in a fluorescence-activated cell sorting tube for analysis. Unstained and Fluorescence Minus One (FMO) control was employed to regulate parameters and to design the gating strategy. CD133 (Prominin-1)-PE antibody (12-1338-43, ThermoFisher Scientific) was used in this study. The fluorescence representing ALDH activity was produced by ALDEFLUOR™ Kit (#01700, Stem Cell Technologies) according to the manual.

### Plasmids, lentivirus production, and transfection

YAP5SA coding sequence was generated by site-directed mutagenesis performed by Q5® Site-Directed Mutagenesis Kit (#E0554, NEW ENGLAND BioLabs). The YAP5SA fragment, wt (wildtype)-YAP-FLAG, and mut (mutation)-YAP-FLAG with depletion of amino acids 261 to 279 were inserted into pCDH-CMV-MCS-EF1-Puro lentivirus plasmid (kindly provided by Professor Hongbin Ji, Shanghai Institutes for Biological Sciences, Chinese Academy of Sciences, Shanghai, China). Two independent shRNAs specifically against YWHAB were inserted into PLKO.1-Puro lentivirus plasmid (also kindly provided by Professor Hongbin Ji). The sequences of shRNAs are listed in Table S3. The 8 × GTIIC promoter region was amplified from an 8xGTIIC-luciferase plasmid (#34615, Addgene) by PCR and inserted into pLV-Promoterless-Firefly luciferase-PGK-Puro (#LVR-1048, Cellomics Technology). Lentivirus was produced by co-transfection of the recombinant plasmids, packaging plasmid (pCMV-dR8.91), and envelope plasmid (pCMV-VSV-G) into HEK293T cells. pLV-Promoterless-Firefly luciferase-PGK-Puro lentivirus was produced by EZ-LentiPACK Lentivirus Packaging System (PAC-10001, Cellomics Technology). The cells were transduced with recombinant lentivirus with polybrene followed by puromycin selection for one week.

### Luciferase reporter assay

The YWHAB knockdown cells and control cells were transfected with pLV-8 × GTIIC-Firefly luciferase-PGK-Puro lentivirus. After one-week selection by puromycin, the cells were transfected with pRL-TK plasmid. After 48-h transfection, the luciferase activities were determined by the Dual-Luciferase® Reporter Assay System (#E1910, Promega). The luciferase signal was normalized by the *Renilla reniformis* luciferase signal.

### Inhibition rate and IC50 determination

The cells were treated with serious concentrations of cisplatin. CCK-8 assay kit (#CK04, Dojindo Molecular Technologies, Tokyo, Japan) was used to determine the cell number. Inhibition rate = 1 – cell viability (absorbance ((treated-blank)/(vehicle-blank)) %). The half-maximal inhibitory concentration (IC50) was calculated by the R package. The IC_50_ values were measured at 72 h after drug treatment (no medium change was performed during measurement).

### Determination of 5mc and m6A levels

Methylated DNA Immunoprecipitation (MeDIP) ChIP Kit and m6A RNA Methylation Assay Kit (Colorimetric) (ab117135 and ab185912, Abcam) were used for determination of 5mc and m6A levels in YWHAB promoter and mRNA, respectively, according to the manual. Briefly, total DNA and RNA were isolated, followed by incubation with capture antibodies. The binding nucleic acids were eluted, followed by quantitative PCR analysis. The primers used are listed in Table S2.

### Molecular docking analysis

The molecular docking analysis (YWHAB-YAP binding) was performed by the online software GRAMM-X (http://gramm.compbio.ku.edu/), Pymol (Version 2.4), and PDBePISA (https://www.ebi.ac.uk/pdbe/pisa/).

### Statistical analysis

Data were represented as mean ± standard deviation from three experiments except where indicated. A paired, two-tailed, student's *t*-test was used to determine the significance of the difference of YWHAB expression between paired OCPM samples. Unpaired, two-tailed, Student's *t*-test and one-way ANOVA were used for regular experiments. Extreme limiting dilution analyses were performed for limiting dilution assay. ∗*p* < 0.05, ∗∗*p* < 0.01, ∗∗∗*p* < 0.001.

## Results

### Proteomic alteration of post-NACT OCPM residual tissues

**Figure 1:**
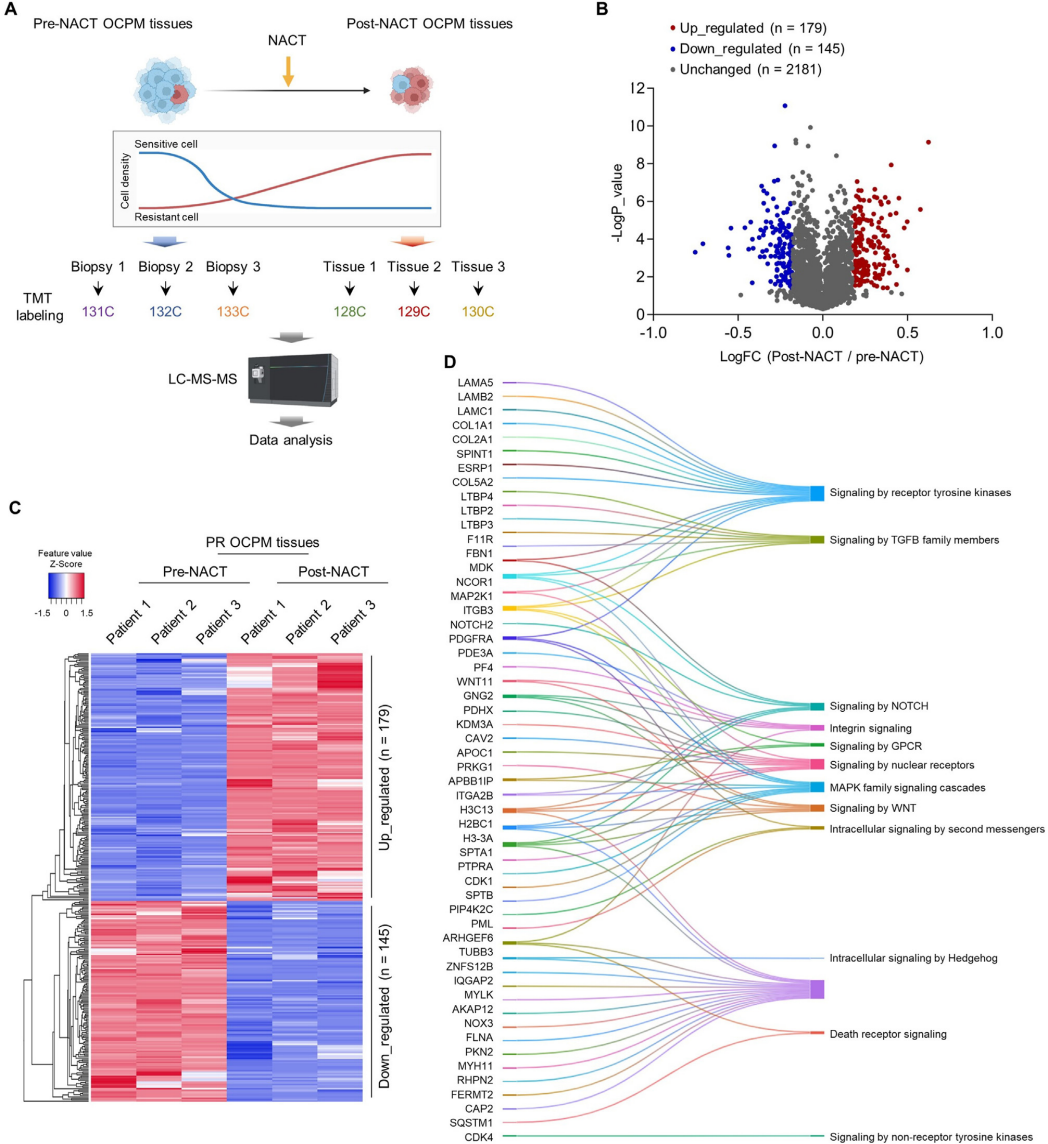
Proteomic alterations in post-NACT OCPM tissues. **(A)** The schematic diagram of the study design. Three paired of pre- and post-NACT PR OCPM tissues from three patients were subjected to proteomic analysis. **(B, C)** Volcano plot (B) and heatmap (C) of the differentially expressed proteins identified in post-NACT OCPM tissues. **(D)** Sankey diagram of the major signaling pathways enriched by the differentially expressed proteins.

The cancer stem cells are usually enriched in residual tumor tissues after chemotherapeutic treatment.^5^^,^^7^ To investigate the mechanisms underlying OCPM stemness maintenance and resistance, three paired pre- and post-NACT OCPM tissues from three patients were subjected to proteomic analysis (Fig. 1A). As shown in Figure S1A–E, the proteomic analysis was successfully performed, reflected by protein coverage, precursor ion tolerance, peptide length, *etc*. In total, 290,991 spectra were detected, 63,816 spectra were matched, and 37,248 peptides and 6094 proteins were identified (Fig. S1F). The differentially expressed proteins were identified by student's *t*-test- and fold change-based filtering. In total, 324 differentially expressed proteins (179 up-regulated and 145 down-regulated) were identified (Fig. 1B, C and Table S4). The results of bioinformatics analysis, including impacted signaling pathways, cellular functions, and metabolisms, are shown in Figure 1D and Table S5; a certain number of previously known and unknown stemness-related proteins and pathways were included.

### Down-regulation of YWHAB in post-NACT OCPM residual tissues identified by tissue microarray analysis

**Figure 2:**
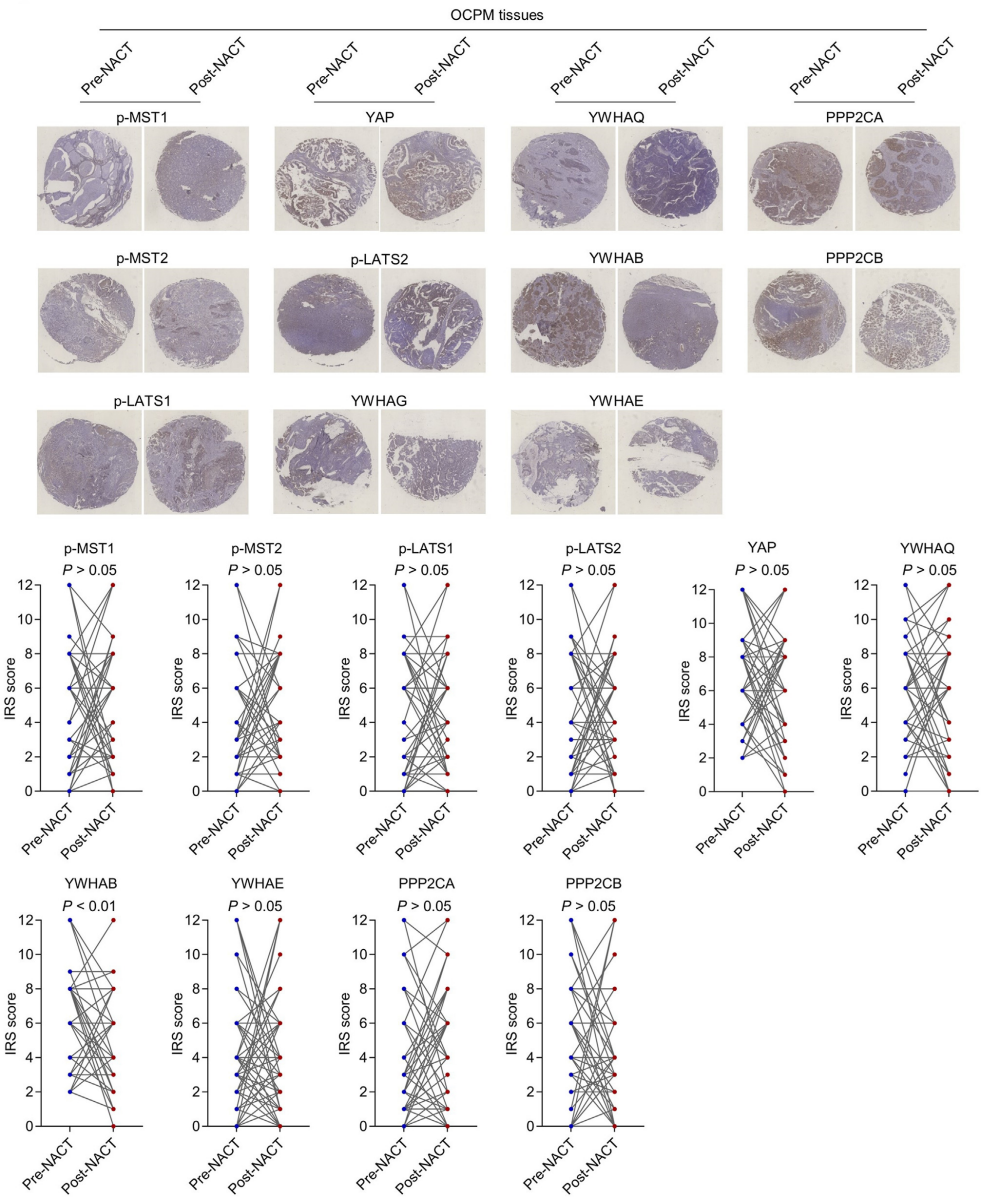
Tissue microarray analysis of the expression of key components in Hippo/YAP signaling in pre- and post-NACT OCPM tissues. A tissue microarray was constructed using 53 pairs of pre- and post-NACT OCPM tissues. The levels of indicated proteins were analyzed by immunohistochemistry. Paired student's *t*-test; ∗*p* < 0.05, ∗∗*p* < 0.01, ∗∗∗*p* < 0.001.

While only three paired tissues from three patients were included in this proteomic study, a considerable number of key regulators may be missed as the result of interpatient tumor heterogeneity.^22^ Meanwhile, Hippo/YAP signaling, essential in cancer stem cells in various cancers,^8^^,^^17^ was surprisingly missing in our data. To announce the shortcomings of our proteomic study and to explore the role of Hippo/YAP signaling in OCPM stemness and resistance, we next performed tissue microarray analysis. The protein levels of several main upstream kinase regulators (active format), p-MST1, p-MST2, p-LATS1, and p-LATS2, transcriptional coactivator, YAP, and two catalytic subunits of PP2A phosphatase, PPP2CA and PPP2CB, were examined. We found that the protein level of YWHAB (a negative regulator of YAP that inhibits YAP activity by leading to YAP cytoplasmic retention via direct binding^8^) was significantly down-regulated in post-NACT OCPM tissues (Fig. 2; *p* < 0.01; *n* = 53; paired student's *t*-test), which indicated that Hippo/YAP signaling may be activated via restriction of YWHAB in post-NACT OCPM and this may be one of the main mechanisms underlying enhanced stemness and resistance in post-NACT OCPM.

### The association between YWHAB restriction and OCPM stemness/resistance

**Figure 3:**
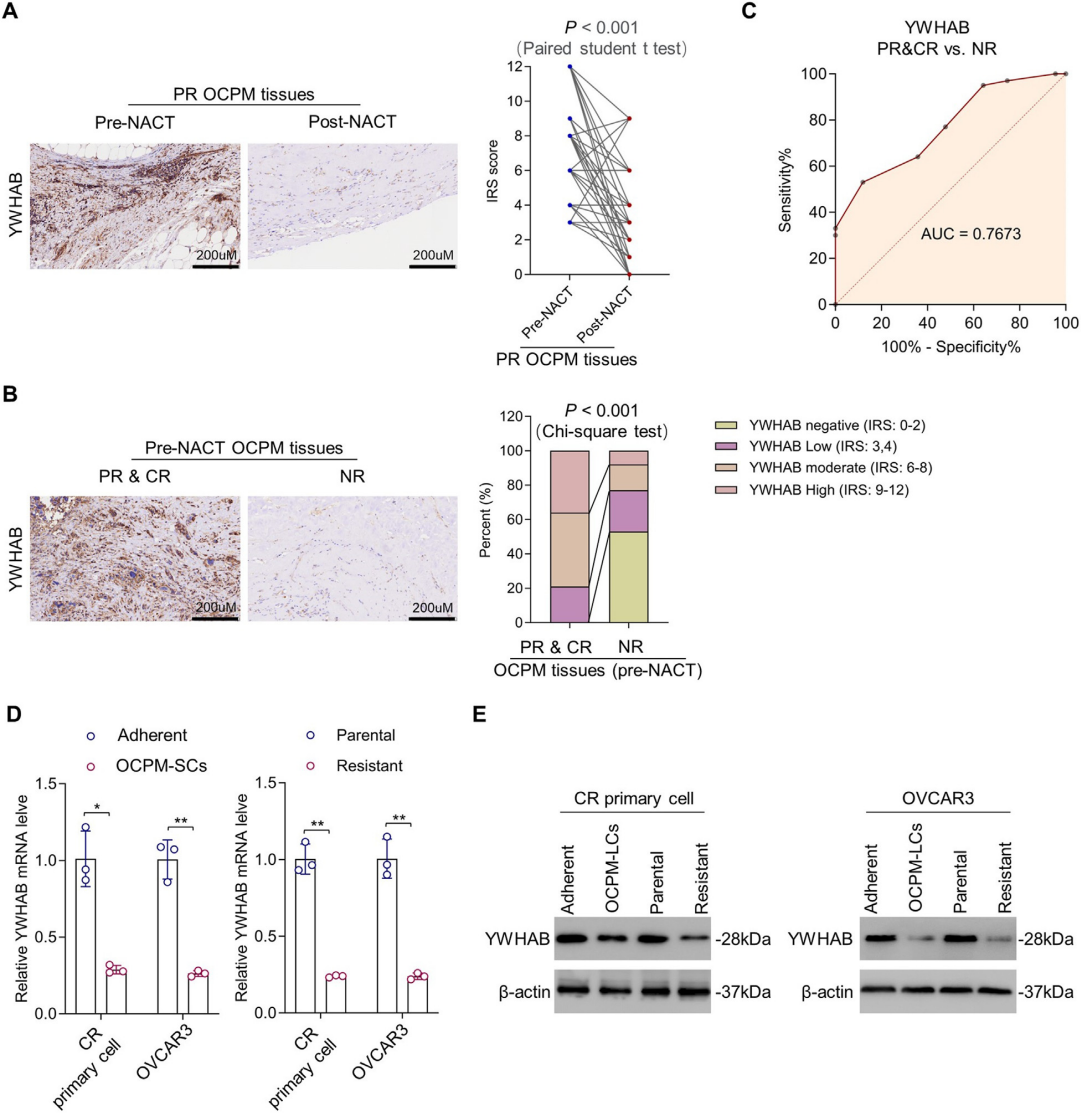
Restricted YWHAB expression is associated with OCPM stemness maintenance and chemoresistance. **(A)** Immunohistochemistry analysis of the protein expression of YWHAB in pre- and post-NACT PR OCPM tissues (paired student's *t*-test; *n* = 53). **(B)** Immunohistochemistry analysis of YWHAB protein level in pre-NACT PR&CR and NR OCPM tissues (Chi-square test; *n* = 67 for PR and CR samples; *n* = 100 for NR samples). **(C)** The receiver-operating-characteristic (ROC) curve describes the diagnostic performance of YWHAB to distinguish NR OCPM. **(D)** The mRNA levels of YWHAB in OCPM-SCs and resistant cells derived from primary CR OCPM and OVCAR3 cells, as well as their parental cells, were determined by quantitative PCR (student's *t*-test; *n* = 3 biological replicates). **(E)** The protein levels of YWHAB in OCPM-SCs and resistant cells derived from primary CR OCPM and OVCAR3, as well as their parental cells, were determined by Western blot assay (student's *t*-test; *n* = 3 biological replicates). ∗*p* < 0.05, ∗∗*p* < 0.01, ∗∗∗*p* < 0.001.

We next performed multiple experiments to confirm the potential association between YWHAB restriction and OCPM stemness/resistance. First, the tissue microarray result about YWHAB down-regulation in post-NACT OCPM tissues was confirmed by immunohistochemistry analysis with paraffin-embedded tissues (Fig. 3A; *n* = 53; *p* < 0.001; paired student's *t*-test). In addition, YWHAB down-regulation was also found in NR OCPM tissues versus PR&CR (Fig. 3B; *n* = 67 for PR and CR samples; 100 for NR samples; *p* < 0.001; Chi-square test); additionally, using YWHAB as a marker efficiently distinguished NR OCPM from PR&CR (Fig. 3C; AUC = 0.7673).

To further investigate the association between YWHAB restriction and OCPM stemness/resistance, we employed primary cells and the OVCAR3 cell line as the models. CR primary OCPM cells with high YWHAB baseline expression and NR primary OCPM cells with low YWHAB baseline expression (shortly named CR or NR primary cells) were first developed; the successful development of these cells was identified by examination of YWHAB expression by quantitative PCR (Fig. S2A; *n* = 3; *p* < 0.001). We next isolated OCPM-SCs and induced cisplatin-resistant cells from CR primary and OVCAR3 cells. As expected, the expression of YWHAB was significantly inhibited in OCPM-SCs and resistant cells, as identified by quantitative PCR (Fig. 3D; *n* = 3; *p* < 0.05 and 0.01 for CR primary and OVCAR3 OCPM-SCs; *p* < 0.01 for both CR primary and OVCAR3 resistant cells, respectively; unpaired, two-tailed, and unequal variance student's *t*-test) and Western blot (Fig. 3E; Fig. S2B; *n* = 3; *p* < 0.05 and 0.01 for OCPM-SCs and resistant cells; unpaired, two-tailed, and unequal variance student's *t*-test). Taken together, these results suggest the association between YWHAB down-regulation and OCPM stemness/resistance.

### Knockdown of YWHAB promotes the stemness and chemoresistance in OCPM

**Figure 4:**
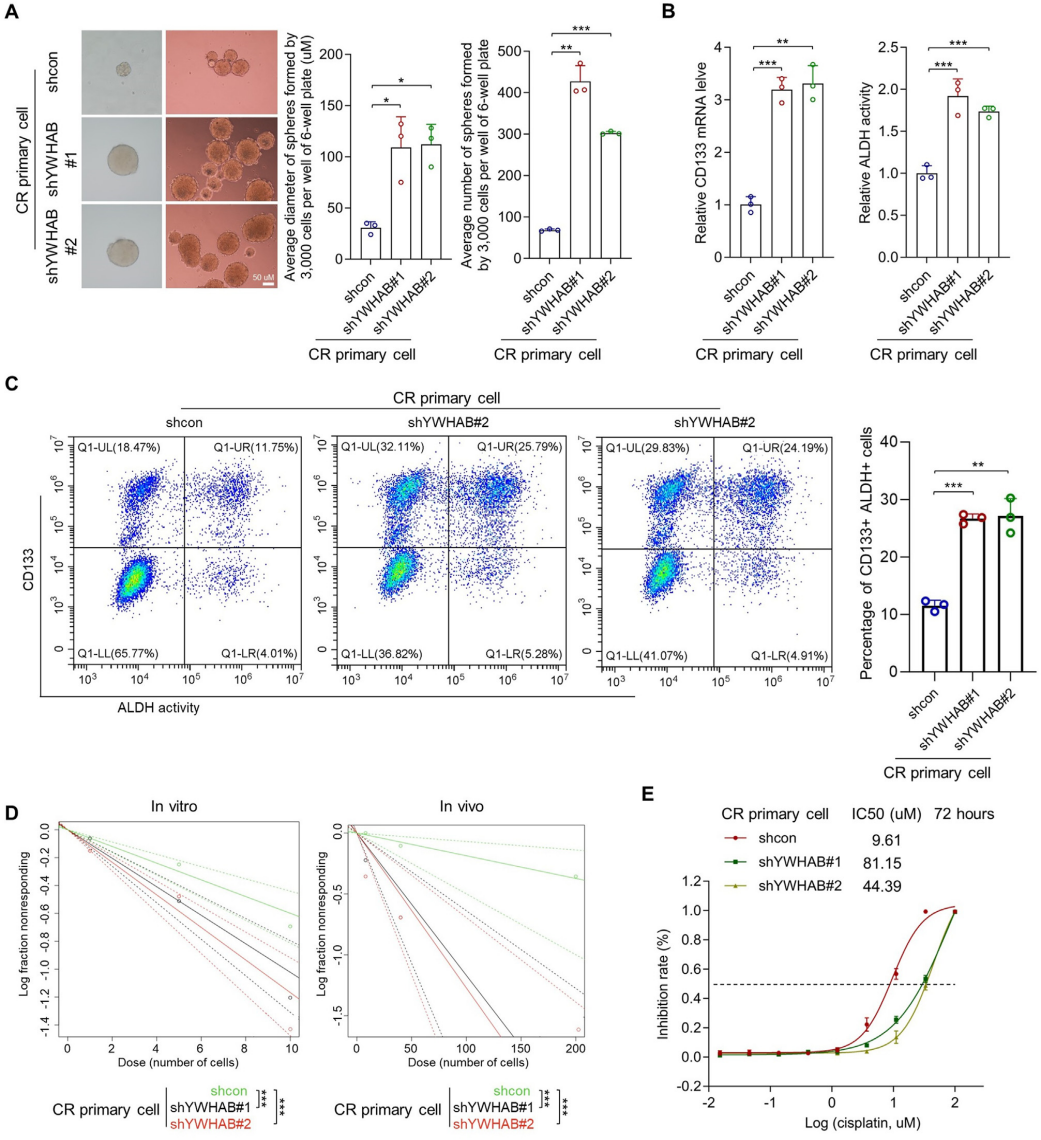
Knockdown of YWHAB promotes stemness and chemoresistance in OCPM. **(A)** The diameter and the number of the spheroids derived from YWHAB-knockdown and control primary CR OCPM cells were tested (one-way ANOVA; *n* = 3 biological replicates). **(B)** The mRNA level of CD133 (left) and the ALDH activity (right) in indicated cells were analyzed by quantitative PCR and ALDH activity assay, respectively (one-way ANOVA; *n* = 3 biological replicates). **(C)** The proportions of CD133^+^ ALDH^+^ cells in indicated cells were analyzed by flow cytometry (one-way ANOVA; *n* = 3 biological replicates). **(D)** The frequencies of *in vitro* sphere-forming cells (left) and *in vivo* tumor-initiating cells (right) in indicated cells were analyzed by limiting dilution assay (extreme limiting dilution analyses, for *in vitro* assay, *n* = 50 biological replicates per group, three groups (1, 5, and 10 cells per well); for *in vivo* assay, *n* = 10 biological replicates per group, three groups (8, 40, and 200 cells per mice)). **(E)** The IC_50_ values of cisplatin in indicated primary CR OCPM cells were tested (*n* = 3 biological replicates). ∗*p* < 0.05, ∗∗*p* < 0.01, ∗∗∗*p* < 0.001.

To confirm the role of YWHAB in OCPM stemness maintenance and resistance, YWHAB was depleted in CR primary cells through lentivirus-mediated gene delivery technology (Fig. S3A). By sphere formation assay, we found that the diameter and the number of the spheres were significantly increased in the cells with YWHAB depletion versus the control cells transfected with empty vectors (Fig. 4A; *n* = 3; *p* < 0.05 for diameter in shYWHAB#1 and shYWHAB#2 cells; *p* < 0.01 and 0.001 for number in shYWHAB#1 and shYWHAB#2 cells; one-way ANOVA test). In addition, ovarian cancer stem cell markers (CD133 mRNA level and ALDH activity) were significantly increased in YWHAB knockdown cells versus control cells (Fig. 4B; *n* = 3; *p* < 0.001 and 0.01 for CD133 mRNA level in shYWHAB#1 and shYWHAB#2 cells, respectively; *p* < 0.001 for ALDH activity in both shYWHAB#1 and shYWHAB#2 cells; one-way ANOVA). This result was also confirmed by flowcytometry, the proportion of CD133^+^ ALDH^+^ cells was significantly increased in YWHAB knockdown cells versus control cells (Fig. 4C; *n* = 3; *p* < 0.001 and 0.01 in shYWHAB#1 and shYWHAB#2 cells, respectively; one-way ANOVA). Moreover, the results from the limiting dilution assay indicated that the frequency of sphere-forming cells *in vitro* and the frequency of tumor-initiating cells *in vivo* were significantly increased in YWHAB knockdown cells (Fig. 4D; Fig. S3B; *p* < 0.001 for *in vitro* sphere-forming cell frequency and *in vivo* tumor-initiating cell frequency in both cells; extreme limiting dilution analysis). Furthermore, YWHAB knockdown cells exhibited more resistance to cisplatin than the control cells (Fig. 4E; *n* = 3; IC50 (72 h) = 81.15 uM and 44.39 uM in YWHAB-knockdown cells versus 9.61 uM in control cells). These results indicate the inhibitory role of YWHAB in stemness maintenance and resistance of OCPM.

### YWHAB depletion promotes YAP activity in OCPM

**Figure 5:**
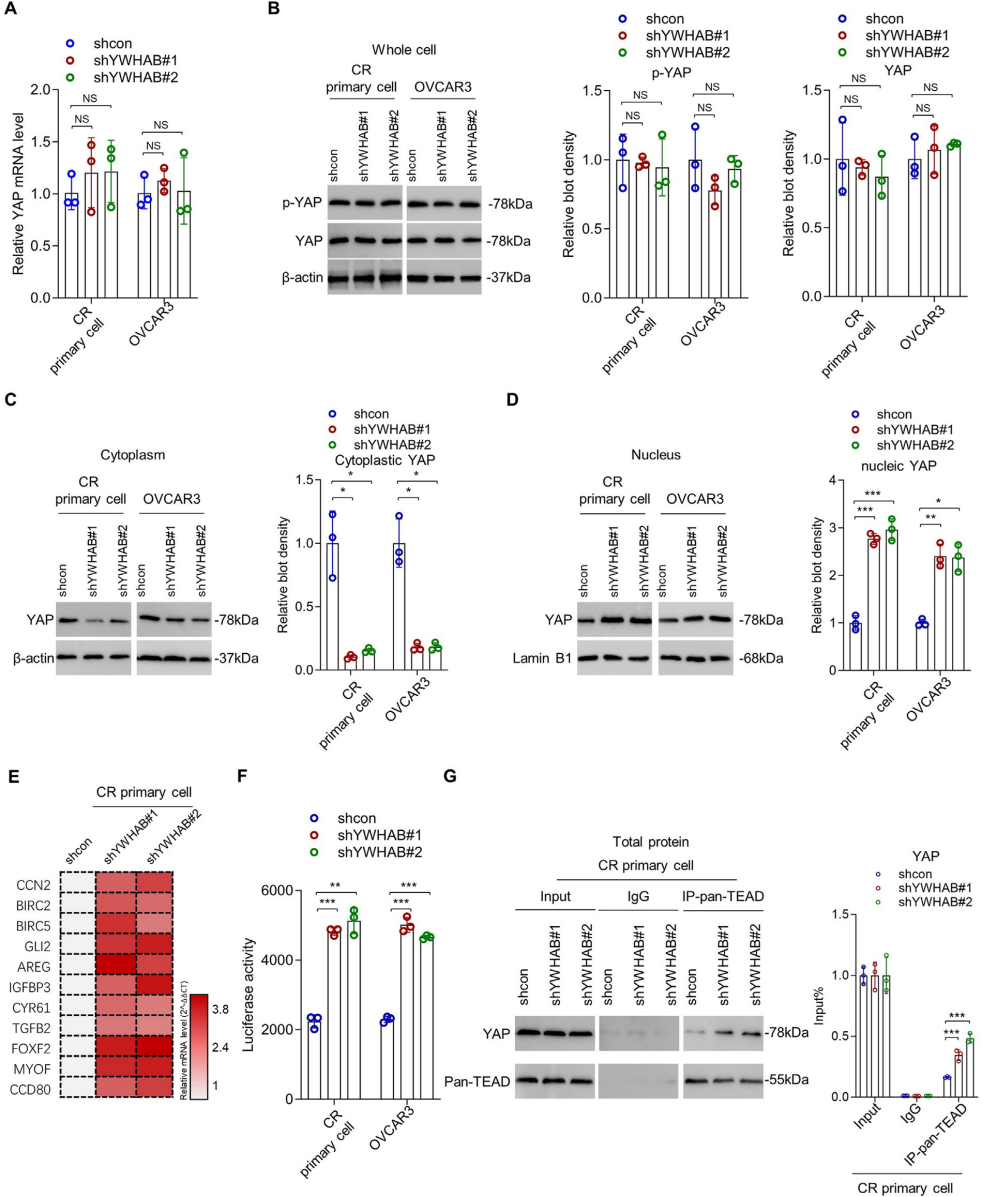
YWHAB depletion promotes YAP activity in OCPM. **(A)** The mRNA levels of YAP in YWHAB-knockdown CR primary OCPM and OVCAR3 cells and their control cells were measured by quantitative reverse-transcription PCR assay (one-way ANOVA; *n* = 3 biological replicates). **(B)** The protein levels of whole cell YAP and phosphorylated YAP in YWHAB-knockdown and control cells were measured by Western blot assay (one-way ANOVA; *n* = 3 biological replicates). **(C, D)** The protein levels of cytoplasmic (C) and nuclear (D) YAP in indicated cells were measured by Western blot (one-way ANOVA; *n* = 3 biological replicates). **(E)** The heatmap representing the relative mRNA levels of indicated YAP target genes in YWHAB-knockdown and control cells. **(F)** The transcriptional activities of YAP in indicated cells were examined by luciferase reporter assay (one-way ANOVA; *n* = 3 biological replicates). **(G)** The abundances of YAP and pan-TEAD immunoprecipitated by pan-TEAD antibody in indicated cells were examined by Western blot (one-way ANOVA; *n* = 3 biological replicates). ∗*p* < 0.05, ∗∗*p* < 0.01, ∗∗∗*p* < 0.001.

Whether YWHAB leads to cytoplasmic retention of phosphorylated YAP in OCPM has not been reported yet. To confirm the role of YWHAB in YAP regulation in OCPM, we first found that the knockdown of YWHAB did not affect the mRNA level of YAP (Fig. 5A; *n* = 3; *p* > 0.05 in all cells; one-way ANOVA) as well as the total protein levels of YAP and phosphorylated YAP (Fig. 5B; *n* = 3; *p* > 0.05 in all cells; one-way ANOVA). However, the abundance of cytoplasmic YAP was significantly reduced in YWHAB knockdown cells versus control cells (Fig. 5C; *n* = 3; *p* < 0.05 in all cells; one-way ANOVA), and conversely, the abundance of nuclear YAP was significantly increased (Fig. 5D; *n* = 3; *p* < 0.001 in both YWHAB knockdown CR primary cells; *p* < 0.01 and 0.05 in shYWHAB#1 and shYWHAB#2 knockdown OVCAR3 cells, respectively; one-way ANOVA). Consistently, the result from the quantitative PCR array showed that the mRNA levels of YAP target genes were significantly increased in YWHAB knockdown CR primary cells versus control cells (Fig. 5E). In addition, the result from the luciferase reporter gene assay indicated that the transcriptional activity of YAP was significantly increased in YWHAB knockdown CR primary and OVCAR3 cells versus control cells (Fig. 5F; *n* = 3; *p* < 0.001 and 0.01 in shYWHAB#1 and shYWHAB#2 knockdown primary cells, respectively; *p* < 0.001 for both YWHAB-knockdown OVCAR3 cells; one-way ANOVA). Moreover, the abundance of YAP immunoprecipitated from TEAD1-4 was significantly increased in YWHAB knockdown CR primary cells versus control cells (Fig. 5G; Fig. S4A; *n* = 3; *p* < 0.001 for both YWHAB-knockdown CR primary cells; one-way ANOVA). Furthermore, the direct binding between YWHAB and YAP in CR primary cells was confirmed by co-immunoprecipitation assays (Fig. S4B). These results support that YWHAB directly binds to YAP and leads to YAP cytoplasmic retention, and thus down-regulation of YWHAB releases YAP from cytoplasm to nucleus, thereby promoting the stemness of OCPM cells.

### The effects of YWHAB knockdown on the stemness are significantly abolished in YAP5SA-overexpressing cells

**Figure 6:**
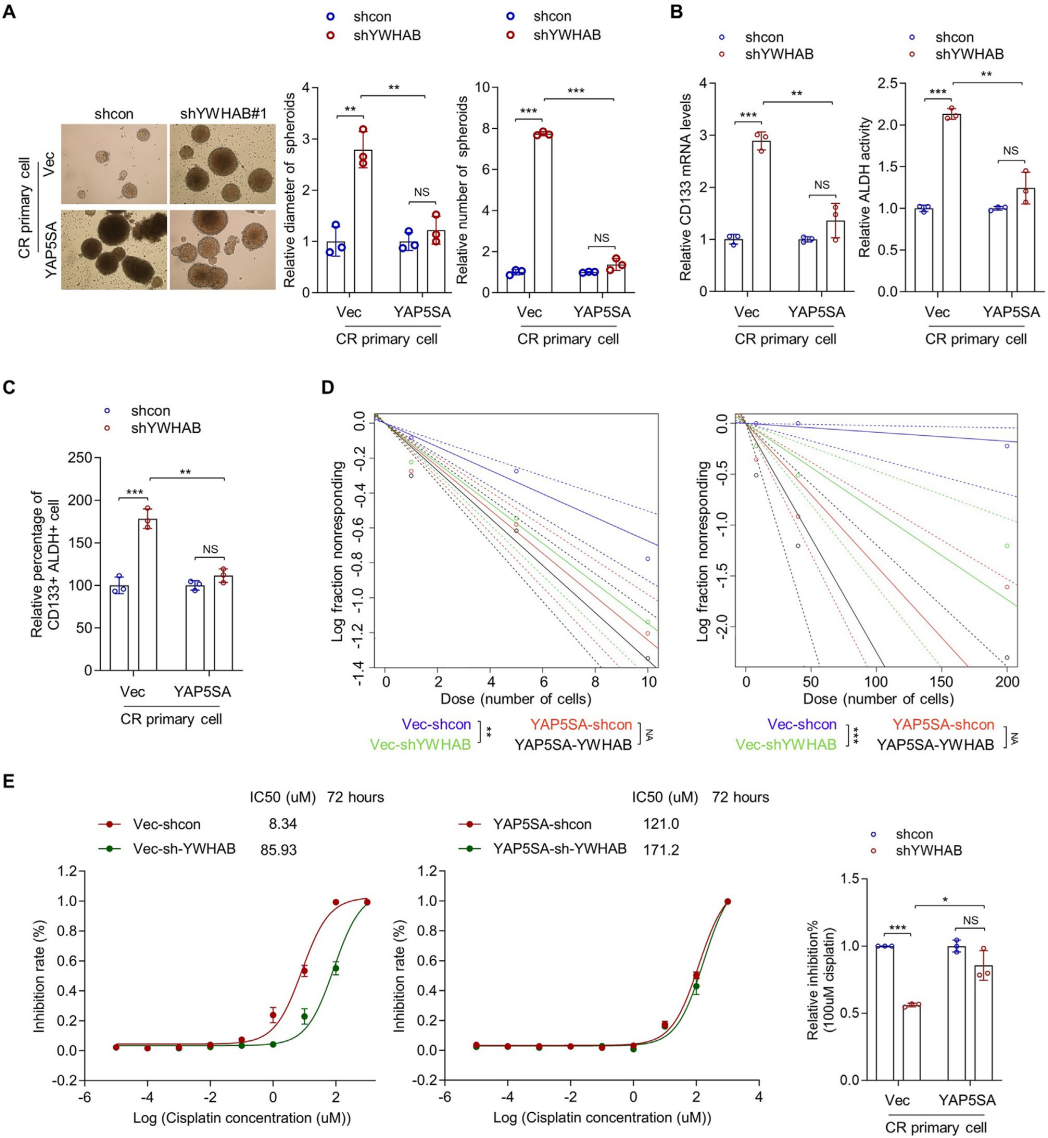
The effects of YWHAB knockdown on the stemness were significantly abolished in YAP5SA-overexpressing cells. **(A)** The diameter and the number of the spheroids derived from YAP5SA-overexpressing and empty-vector control cells transfected with shRNAs against YWHAB and control shRNAs were measured (one-way ANOVA; *n* = 3 biological replicates). **(B)** The mRNA level of CD133 (left) and the ALDH activity (right) in indicated cells were determined by quantitative PCR and ALDH activity assay, respectively (one-way ANOVA; *n* = *3* biological replicates). **(C)** The proportion of CD133^+^ ALDH^+^ cells in indicated cells was analyzed by flow cytometry (one-way ANOVA; *n* = 3 biological replicates). **(D)** The frequencies of *in vitro* sphere-forming cells (left) and *in vivo* tumor-initiating cells (right) in indicated cells were analyzed by limiting dilution assay (extreme limiting dilution analyses; for *in vitro* assay, *n* = 50 biological replicates per group, three groups (1, 5, and 10 cells per well); for *in vivo* assay, *n* = 10 biological replicates per group, three groups (8, 40, and 200 cells per mice)). **(E)** The IC_50_ values of cisplatin in indicated cells were tested by cell viability assay (*n* = 3 biological replicates). The inhibition rate of 100 uM cisplatin in indicated cells was provided. ∗*p* < 0.05, ∗∗*p* < 0.01, ∗∗∗*p* < 0.001.

To further confirm the role of YWHAB down-regulation in OCPM stemness maintenance, we performed a rescue experiment by employing YAP5SA-overexpressing CR primary cells, in which the Hippo/YAP signaling was constitutively activated by the overloaded constitutively active YAP (YAP5SA, mutation of all five serines to alanines (5SA) and so is free from the inhibitory effects of almost all regulators, including kinases in Hippo signaling as well as YWHA proteins and other kinases or phosphates^23^). YAP5SA-overexpressing YWHAB-knockdown cells were established (Fig. S5A), and as expected, no difference in the level of nuclear YAP was observed in YAP5SA-overexpressing YWHAB-knockdown cells (Fig. S5B), which indicated that the YAP activity was free from YWHAB knockdown and so the cell models for this rescue experiment was successfully established. Consistently, the effects of YWHAB knockdown on the stemness and resistance of CR primary cells were significantly abolished, reflected by spheroid diameter and number (Fig. 6A), CD133 expression (Fig. 6B, left), ALDH activity (Fig. 6B, right), the proportion of CD133^+^ ALDH^+^ cells (Fig. 6C), the frequency of sphere-forming (Fig. 6D, left; Fig. S5C) and tumor-initiating cells (Fig. 6D, right; Fig. S5C), IC50 value (Fig. 6E; *n* = 3; IC50 = 8.34 uM and 85.93 uM in vec-shcon and vec-shYWHAB#1 cells, respectively; IC50 = 121.0 uM and 171.2 uM in YAP5SA-shcon and YAP5SA-shYWHAB#1 cells, respectively), and cisplatin (100 uM) inhibition rate (Fig. 6E). These results support that YAP5SA overexpression abolishes the effect of YWHAB knockdown on OCPM stemness and confirm that YWHAB knockdown promotes OCPM stemness and resistance through YAP.

### SH3 domain in YAP is critical for YAP-YWHAB binding

**Figure 7:**
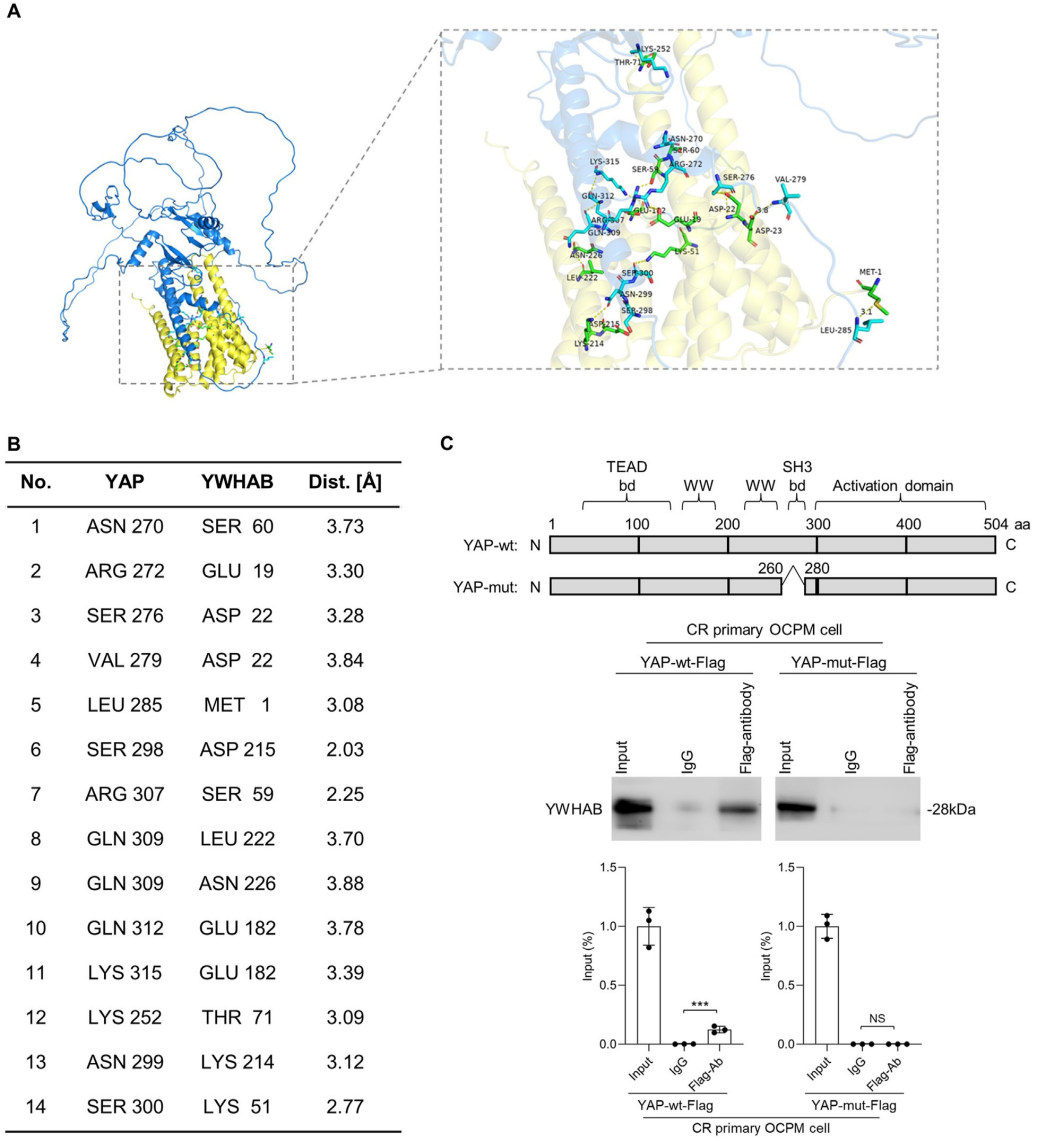
SH3 domain in YAP is critical for YAP-YWHAB binding. **(A)** The binding between YWHAB and YAP was predicted by *in silico* analysis. **(B)** The binding amino acids of the YWHAB/YAP complex predicted by *in silico* analysis. **(C)** The bindings between YWHAB and wt-YAP-FLAG and mut-YAP-FLAG with depletion of amino acids 261 to 279 were analyzed by co-immunoprecipitation assay. One-way ANOVA; *n* = 3; ∗*p* < 0.05, ∗∗*p* < 0.01, ∗∗∗*p* < 0.001.

The key domains for YAP-YWHAB binding have remained elusive. As shown in Figure 7A, the binding between YAP and YWHAB was predicted by *in silico* analysis; we found that YWHAB was embedded in YAP, and SH3 binding domain^24^ in YAP would be important for the binding (Fig. 7A, B). We thus developed stable cells transfected with wt-YAP-FLAG and mut-YAP-FLAG with depletion of amino acids 261 to 279 plasmids (Fig. 7C), followed by co-immunoprecipitation assay. As shown in Figure 7C, the YWHAB-YAP binding was disrupted by the disruption of the SH3 binding domain in YAP, which suggests that the SH3 domain in YAP is critical for YAP-YWHAB binding.

## Discussion

**Figure 8:**
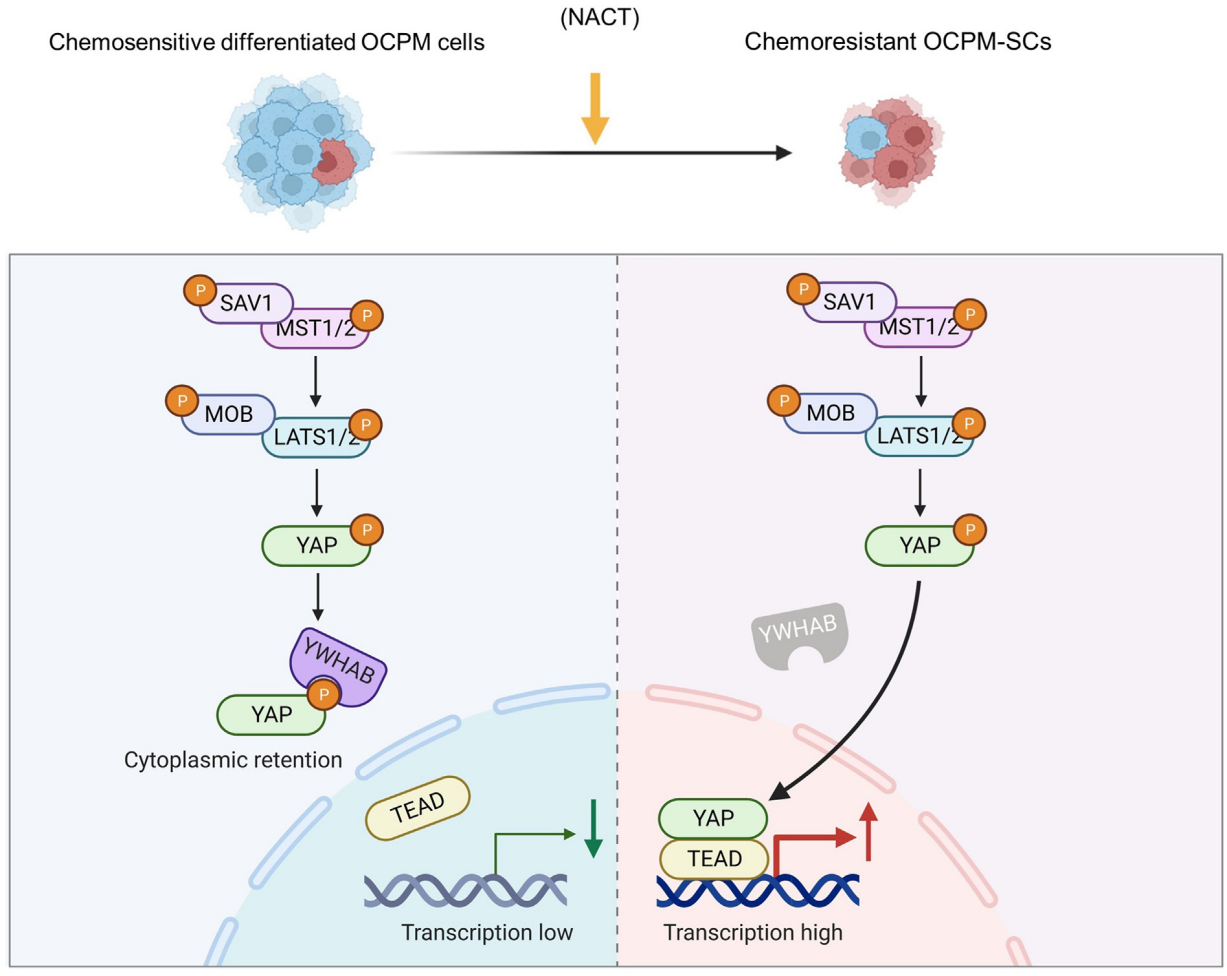
The schematic diagram of the mechanism underlying OCPM stemness and resistance maintenance mediated by YWHAB restriction. Down-regulation of YWHAB in OCPM-SCs attenuates YAP cytoplasmic retention and promotes YAP nuclear accumulation, thereby strengthening YAP-mediated OCPM stemness and resistance.The figure was created using BioRender (www.biorender.com).

In this study, we explored the proteomic alteration in post-NACT OCPM tissues by tandem mass tag-based proteomic study. Subsequently, we announced the shortcomings of this proteomic study resulting from a limited number of samples, performed tissue microarray-based assay, and revealed a novel mechanism underlying OCPM stemness maintenance and resistance that restricted expression of YWHAB attenuated YAP cytoplasmic retention and promoted YAP nuclear accumulation, thereby activating YAP-mediated stemness maintenance and resistance in OCPM (Fig. 8).

Liquid chromatography mass spectrometry-based proteomic study is consumable that usually limits the patient number recruited.^25^ In addition, the repetitiveness of the proteins identified and quantified by liquid chromatography-mass spectrometry-based analysis is limited to some extent.^25^ In this study, we first identified proteomic alteration of post-NACT in three patients, and revealed a considerable number of previously unknown targets, such as NCOR1, H3C13, MDK, and H2BC1 in NOTCH pathway, GNG2, PRKG1, and WNT11 in Wnt pathway, SPTA1, PTPRA, ITGB3, and SPTB in MAPK pathway (Fig. 1). While, the role of Hippo/YAP signaling was surprisingly not included, which was subsequently explored by tissue microarray-based study (Fig. 2), and so announced the shortcomings of liquid chromatography-mass spectrometry-based proteomic study as the result of the limited number of the samples. While, our liquid chromatography-mass spectrometry-based proteomic study would be also useful for further exploring the novel mechanisms underlying resistant OCPM, at least for exploring the heterogenicity of OCPM and context-dependent functions of regulatory proteins.

YWHAB is a member of the YWHA family. YWHA proteins are highly conserved and bind to various proteins.^26^ Thus, the roles of YWHA family proteins are context-dependent. For example, Miao and colleagues reported that YWHAE-driven cytoplasmic retention of YAP suppressed tumor progression and lymphangiogenesis in clear-cell renal cell carcinoma,^27^ while, Yang and colleagues showed that YWHAE promoted the proliferation, metastasis, and chemoresistance in breast cancer cells.^28^ YWHAB was reported to execute stimulatory roles in the colon,^29^ cervical,^30^ and lung cancer cells.^31^ Considering the interpatient heterogenicity and the context-dependent role of YWHA proteins, more clinical samples and primary cells should be employed for a deeper understanding of the roles of YWHAB in OCPM.

We also explored the mechanism underlying the down-regulation of YWHAB in OCPM-SCs. Epigenetic regulation plays a fundamental role in hereditably regulating gene expression.^32^ We thus examined the changes in 5mc methylation in YWHAB promotor as well as m6A in YWHAB mRNA. We found that the 5mc level in the YWHAB promotor was significantly decreased in OCPM-SCs versus adherent cells (Fig. S6A), while, no difference in m6A in YWHAB mRNA was observed (Fig. S6B). These results indicated that changing the 5mc level may be involved in restricted YWHAB in OCPM-SCs.

In conclusion, our results revealed that restriction of YWHAB-mediated YAP cytoplasmic retention is a novel mechanism underlying OCPM stemness maintenance. The key domain in YAP for YWHAB-YAP binding was revealed. Our findings suggest that YAP inhibitors would be an effective therapeutic option for suppressing the OCPM stemness caused by YWHAB restriction.

## Ethics declaration

All the experiments were conducted in accordance with the Declaration of Helsinki and the Good Clinical Practice guidelines. The study protocol and any amendments were approved by the Institutional Review Board of the Cancer Hospital of Dalian University of Technology (ethics number: 2021G0319). Written informed consent was received from all patients.

## Funding

This research was partially funded by the 10.13039/501100001809National Natural Science Foundation of China (No. 82103056), the Liaoning Province Science and Technology Plan Project (China) (No. 2022JH2/101300045, 2023-MS-060), the Fundamental Research Funds for the Central Universities (China) (No. LD202208), the Liaoning Cancer Hospital and Institute, Dalian University of Technology “Medical Industrial Interdisciplinary Research Fund” (No. LD2023028), the Chongqing Science & Technology Commission of China (No. CSTB2022NSCQ-MSX1413, CSTB2023TIAD-KPX0052), and the Shanghai Hongkou District Health Commission of China (No. Hongwei 2101-01).

## Data availability

The mass spectrometry proteomics data are available via ProteomeXchange with the identifier PXD044243.

## CRediT authorship contribution statement

**Chang Liu:** Formal analysis. **Lei Shi:** Investigation, Methodology. **Zijun Meng:** Formal analysis. **Manlin Zhang:** Formal analysis. **Zhiqi Zhang:** Formal analysis, Funding acquisition, Investigation. **Yunzhe Li:** Formal analysis. **Kaiwen Du:** Data curation, Formal analysis. **Muyao Yang:** Data curation, Formal analysis. **Lin Qiu:** Data curation, Formal analysis. **Jing Feng:** Data curation, Formal analysis. **Yuchen He:** Data curation, Formal analysis. **Jiayun Liu:** Data curation, Formal analysis, Investigation. **Hua Zhang:** Funding acquisition, Investigation, Methodology. **Hongbin Zhang:** Conceptualization, Supervision. **Tingyuan Lang:** Conceptualization, Data curation, Formal analysis, Funding acquisition, Investigation, Supervision, Writing – original draft, Writing – review & editing. **Zhuo Yang:** Conceptualization, Funding acquisition, Investigation, Methodology, Project administration, Writing – original draft, Writing – review & editing.

## Conflict of interests

All authors have no interests to declare.
