# Supplementary material for: Restriction of YWHAB-mediated YAP cytoplasmic retention is a novel mechanism underlying stemness maintenance and chemoresistance in ovarian cancer peritoneal metastasis
Source: Genes Dis. 2025 Jan 8;12(5):101519. doi: 10.1016/j.gendis.2025.101519 (PMC12221726; doi:10.1016/j.gendis.2025.101519)
Supplement: Multimedia component 1 [file mmc1.docx]

Supplementary materials

**Restriction of YWHAB-mediated YAP cytoplasmic retention is a novel mechanism underlying stemness maintenance and chemoresistance in ovarian cancer peritoneal metastasis**

Chang Liu ^a,1^, Lei Shi ^b,1^, Zijun Meng ^c, d, 1^, Manlin Zhang ^e, 1^, Zhiqi Zhang ^f^, Yunzhe Li ^g^, Kaiwen Du ^g^, Muyao Yang ^h^, Lin Qiu ^a^, Jing Feng ^a^, Yuchen He ^i^, Jiayun Liu ^i^, Hua Zhang ^g^, Hongbin Zhang ^j,^ ***, Tingyuan Lang ^c,^ **, Zhuo Yang ^a,^ *

^a^ Department of Gynaecology, Cancer Hospital of Dalian University of Technology; Cancer Hospital of China Medical University; Liaoning Cancer Hospital & Institute, Shenyang, Liaoning 110001, The People's Republic of China.

^b^ Hematology and oncology department The People's Hospital of Tongliang District, Chongqing 402560, The People's Republic of China.

^c^ Reproductive Medicine Center, The First Affiliated Hospital of Chongqing Medical University, Chongqing 400016, The People's Republic of China.

^d^ Chongqing Key Laboratory of Translational Medical Research in Cognitive Development and Learning and Memory Disorders, Ministry of Education Key Laboratory of Child Development and Disorders, National Clinical Research Center for Child Health and Disorders, China International Science and Technology Cooperation Base of Child Development and Critical Disorders, Children’s Hospital of Chongqing Medical University, Chongqing 400015, The People's Republic of China.

^e^ Department of Obstetrics and Gynecology, Shengjing Hospital of China Medical University, Shenyang, Liaoning 110136, The People's Republic of China.

^f^ Department of General Surgery, Shanghai Fourth People’s Hospital, School of Medicine, Tongji University, Shanghai 200434, The People's Republic of China.

^g^ Department of Gynaecology and Obstetrics, The First Affiliated Hospital of Chongqing Medical University, Chongqing 400016; Department of Gynecology, The First Branch of The First Affiliated Hospital of Chongqing Medical University, Chongqing, 400042; State Key Laboratory of Maternal and Fetal Medicine of Chongqing, Chongqing Medical University, Chongqing 400016; The People's Republic of China.

^h^ College of Bioengineering, Chongqing University, Chongqing 400044, The People's Republic of China.

^I^ Department of Clinical Laboratory, Xijing Hospital, Fourth Military Medical University, Xi'an 710032, The People's Republic of China.

^j^ Department of Hematology, The First Affiliated Hospital of Chongqing Medical University, Chongqing 400016, The People's Republic of China.

*Corresponding author. Department of Gynaecology, Cancer Hospital of Dalian University of Technology; Cancer Hospital of China Medical University; Liaoning Cancer Hospital & Institute, Shenyang, Liaoning 110001, The People's Republic of China.

**Corresponding author. Reproductive Medicine Center, The First Affiliated Hospital of Chongqing Medical University, Chongqing 400016, The People's Republic of China.

***Corresponding author. Department of Hematology, The First Affiliated Hospital of Chongqing Medical University, Chongqing 400016, The People's Republic of China.

*E-mail addresses*: [zhanghongbin@hospital.cqmu.edu.cn](mailto:zhanghongbin@hospital.cqmu.edu.cn) (H. Zhang), [langtingyuan@hospital.cqmu.edu.cn](mailto:langtingyuan@hospital.cqmu.edu.cn) (T. Lang), [yangzhuo@cancerhosp-ln-cmu.com](mailto:yangzhuo@cancerhosp-ln-cmu.com) (Z. Yang)

^1^ These authors have contributed equally to this work.

**Supplementary Figures**


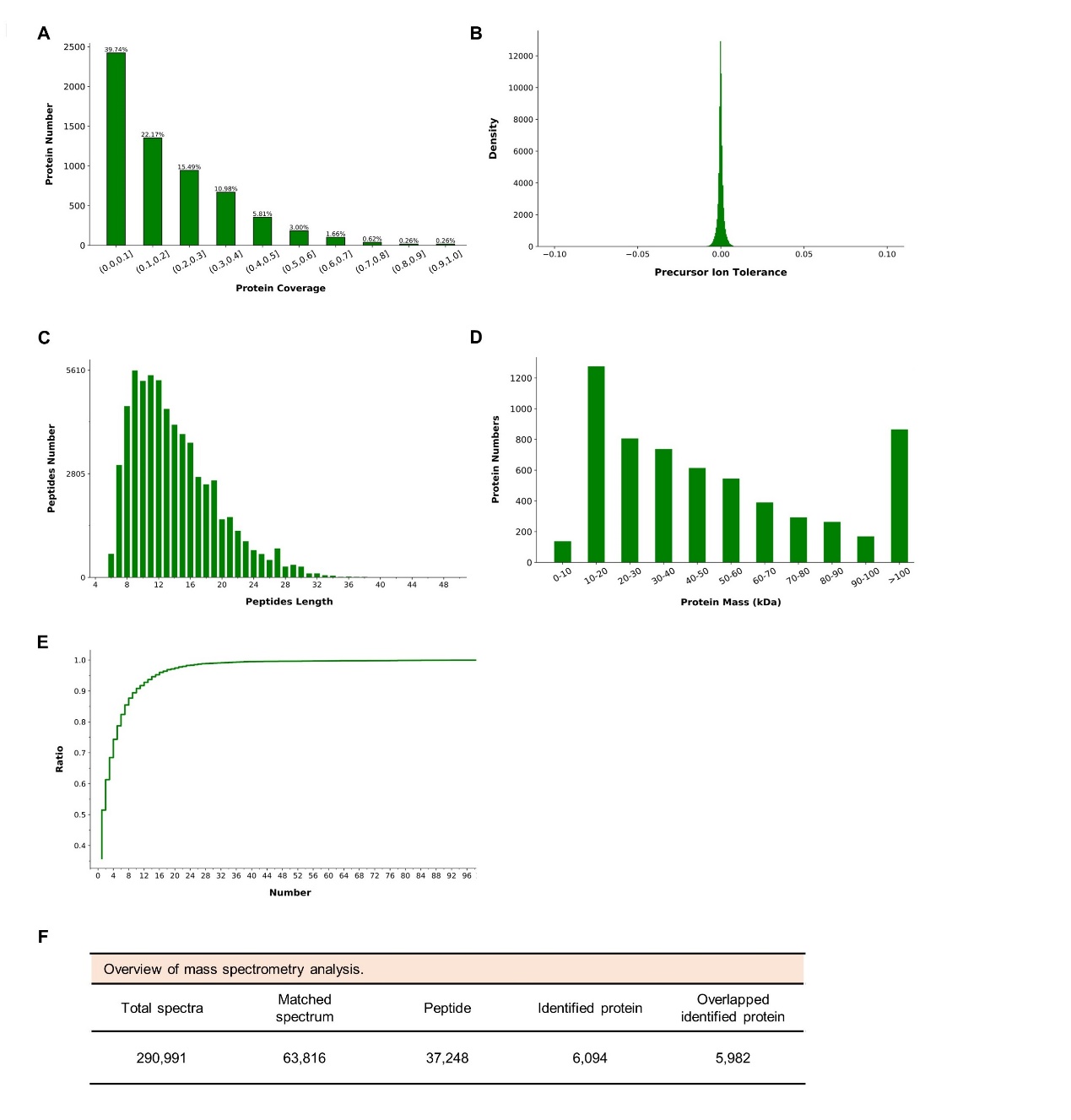


**Supplementary Figure S1.** **Quality control of the proteomic analysis result (related to Figure 1)**. The quality of the proteomic study was assessed by protein coverage (**A**), precursor ion tolerance (**B**), peptides length (**C**), protein mass (**D**), and number (**E**). (**F**) Overview of mass spectrometry analysis.


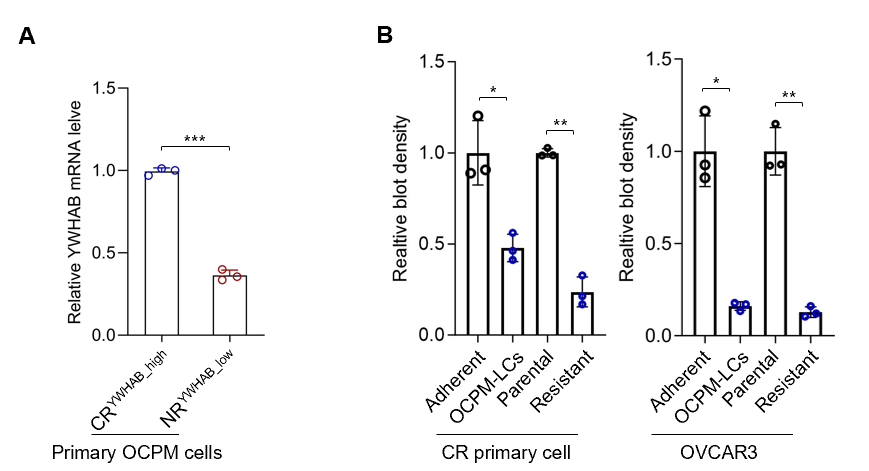


**Supplementary Figure S2. Restricted YWHAB expression is associated with OCPM stemness maintenance and chemoresistance (related to Figure 3).** (**A**) The mRNA level of YWHAB in primary cells isolated from pre-NACT CR (with high YWHAB baseline expression) and NR (with low YWHAB baseline expression) tissues were determined by qPCR (student’s *t*-test, *n* = 3 biological replicates). (**B**) Statistics for Figure 3E. *p* < 0.05, ***p* < 0.01, ****p* < 0.001.


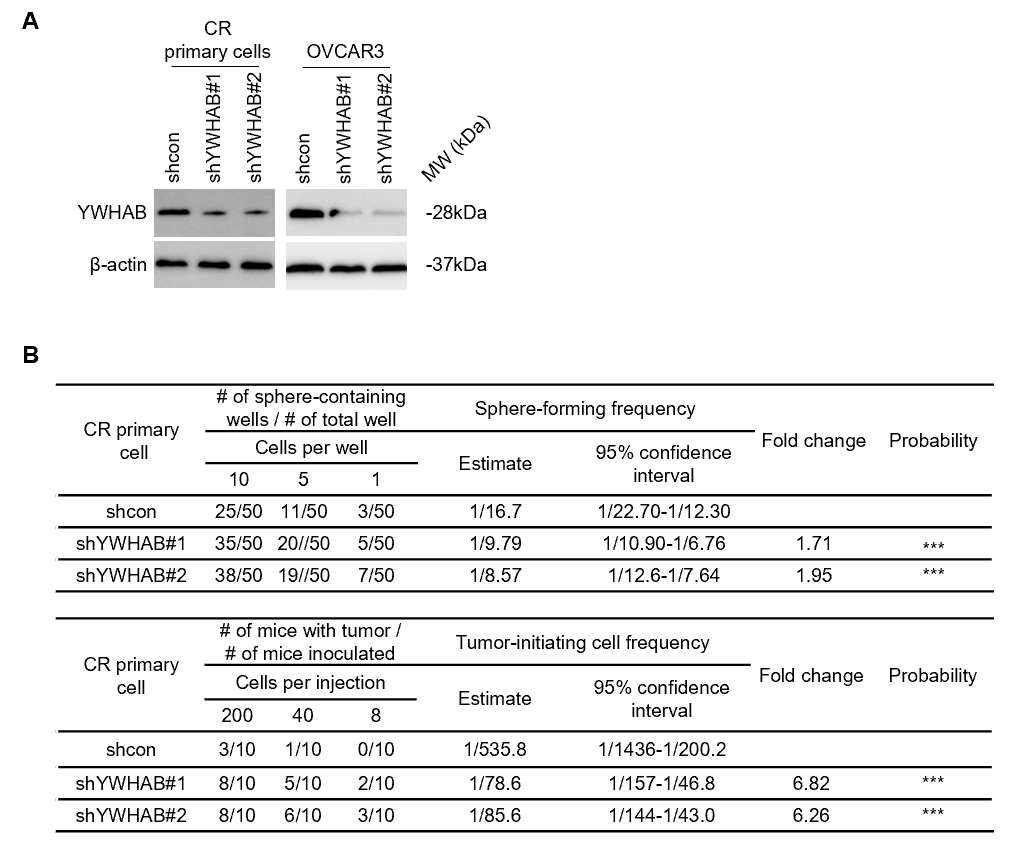


**Supplementary Figure S3. YWHAB-knockdown decreases the frequency of sphere-forming and tumor-initiating cells of OCPM cells (related to Figure 4).** (**A**) Characterization of YWHAB-knockdown primary pCR OCPM cells and OVCAR3 cells. The protein levels of YWHAB in indicated cells were measured by western blot assay. (**B**) YWHAB-knockdown decreases the frequency of sphere-forming and tumor-initiating cells of OCPM cells. The frequencies of sphere-forming and tumor-initiating cells in indicated cells were measured by limiting dilution assay.


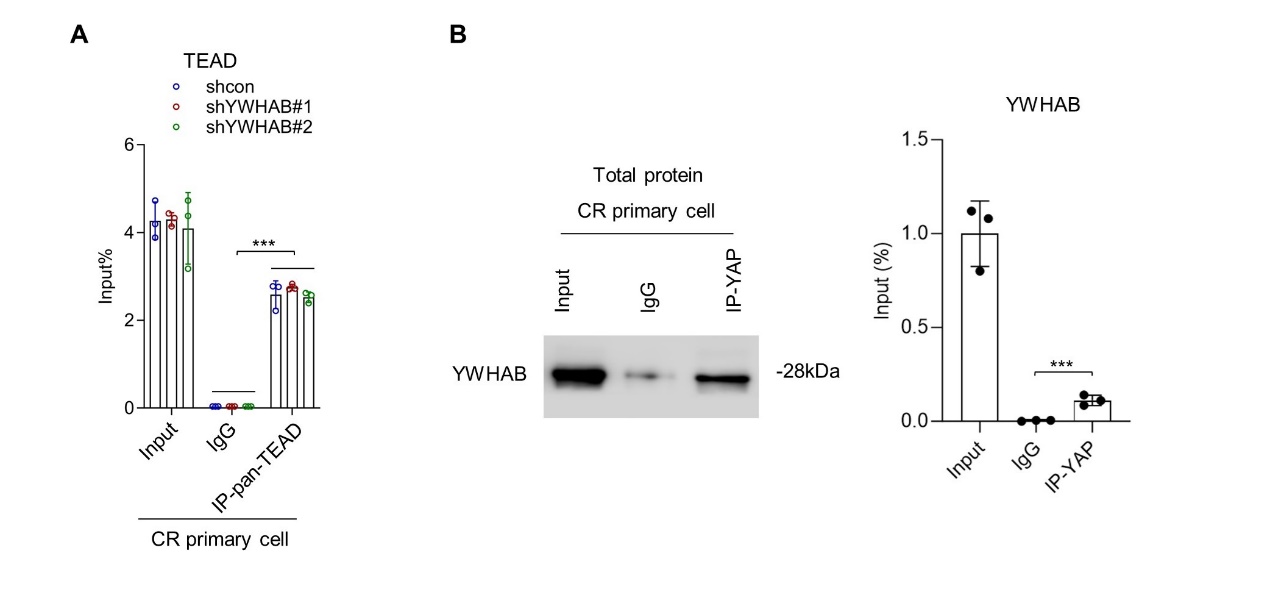


**Supplementary Figure S4. YWHAB depletion promotes YAP activity in OCPM. (related to Figure 5).** (**A**) Statistic of the western blot result representing the abundances of pan-TEAD immunoprecipitated by pan-TEAD antibody in indicated cells (one-way ANOVA, *n* = 3). (**B**) The binding between YWHAB and YAP was analyzed by co-immunoprecipitation assay (one-way ANOVA, *n* = 3). *p* < 0.05, ***p* < 0.01, ****p* < 0.001.


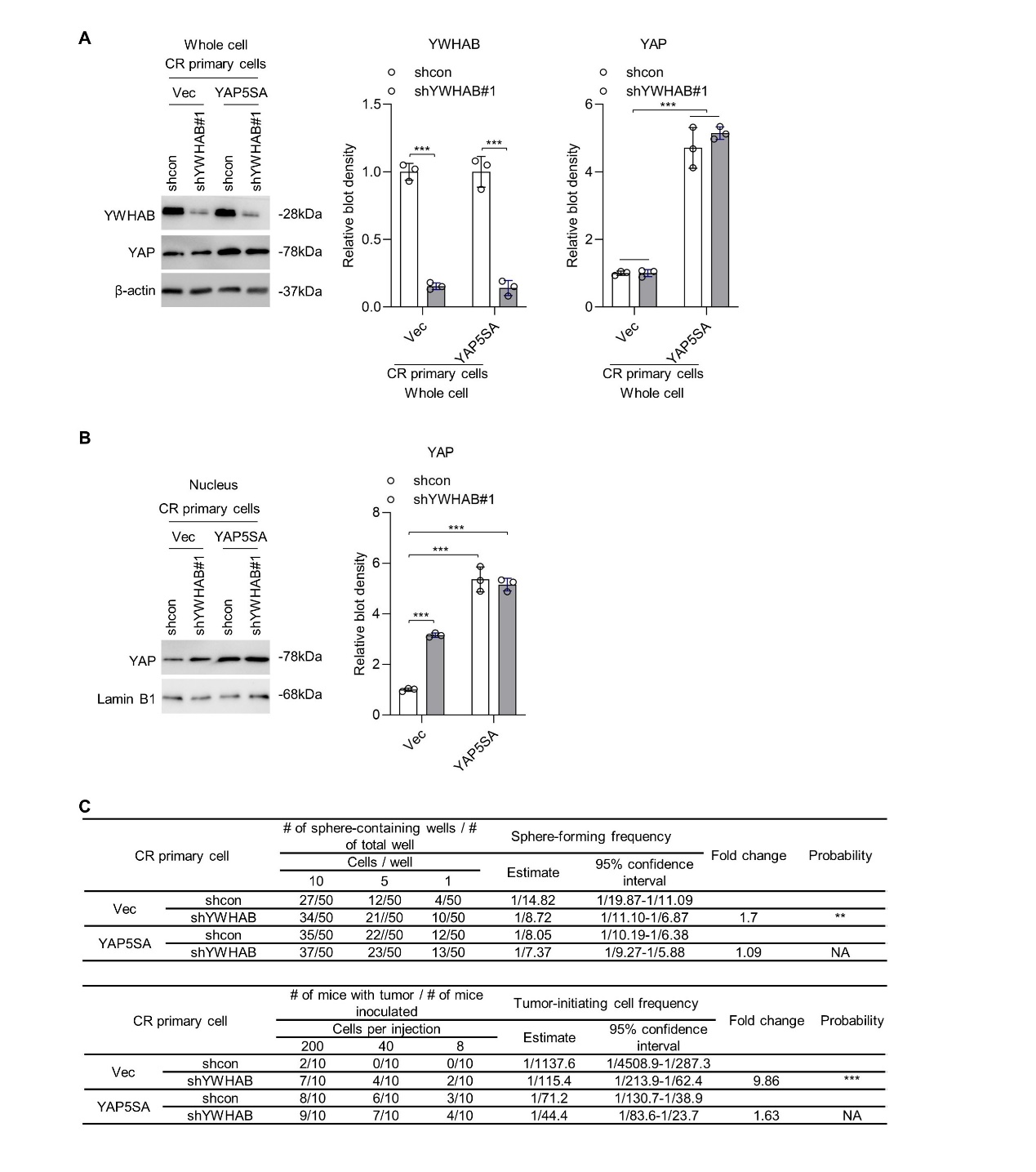


**Supplementary Figure S5. The effects of YWHAB knockdown on the stemness was significantly abolished in YAP5SA-overepxressing cells (related to Figure 6).** (**A**) Characterization of YWHAB-knockdown YAP5SA-overxpressing cells by western blot. The protein expression of YWHAB and YAP in indicated cells was tested. Student’s *t*-test and one-way ANOVA, *n* = 3. (**B**) The protein levels of nuclear YAP in indicated cells were examined by western blot. One-way ANOVA, *n* = 3. (**C**) YAP5SA overexpression abolishes the effect of YWHAB knockdown on the frequency of sphere-forming and tumor-initiating cells of OCPM cells. The frequencies of sphere-forming and tumor-initiating cells in indicated cells were measured by limiting dilution assay (ELDA examination). *p* < 0.05, ***p* < 0.01, ****p* < 0.001.


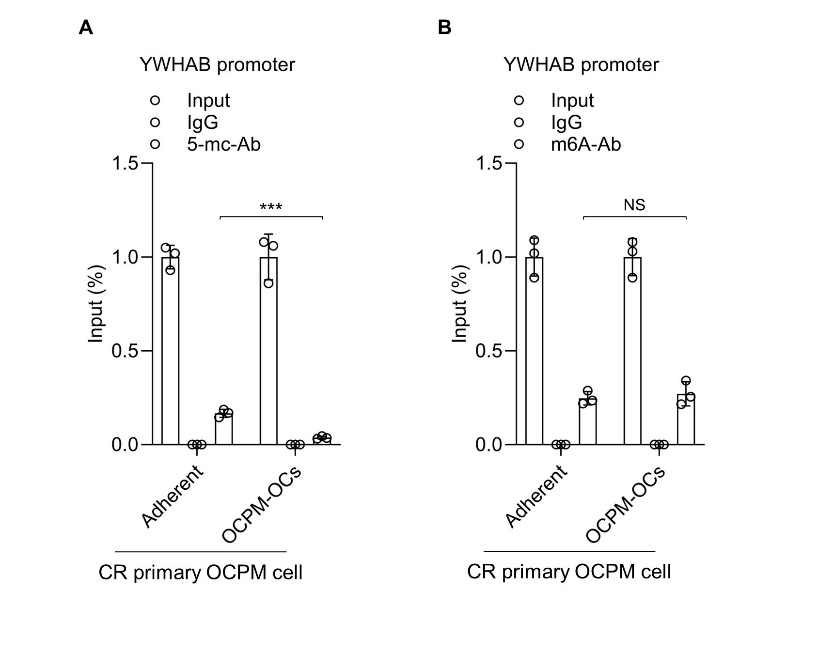


**Supplementary Figure S6. Alteration of 5-mc methylation level in YWHAB promoter in OCPM-SCs.**

(**A,B**) Examination of 5-mc methylation level in YWHAB promoter and m6A methylation level in YWHAB mRNA in indicated cells by ChIP-qPCR. One-way ANOVA, n = 3. *p* < 0.05, ***p* < 0.01, ****p* < 0.001.

**Supplementary Tables**

| **Supplementary TableS1. Clinical characteristics of ovarian cancer patients.** | | | | |  |  |
| --- | --- | --- | --- | --- | --- | --- |
|  | Proteomic study (n = 3, paired) | | Immunohistochemistry study (n = 167) | Primary culture | | |
|  | Pre-NACT | Post-NACT |  | 1 | 2 | 3 |
| Age (years) | | | | |  |  |
| mean ± SD | 57 ± 7.48 | | 60 ± 10.52 | 65 | 63 | 61 |
| Histology (n, (%)) | | | | |  |  |
| HGSOC | 3 (100%) | | 167 (100%) | 1 (100%) | 1 (100%) | 1 (100%) |
| Response to NACT (n, (%)) | | | | |  |  |
| Complete | 0 (0%) | | 14 (8.38%) | 1 (100%) | 0 (0%) | 0 (0%) |
| Partial | 3 (100%) | | 53 (31.74%) | 0 (0%) | 1 (100%) | 0 (0%) |
| No | 0 (0%) | | 100 (59.88%) | 0 (0%) | 0 (0%) | 1 (100%) |
| Median survival (years) | | | | |  |  |
| Median (range) | 2.3 (1.5 – 3.1) | | 1.85 (1.1 – 5.3) | 5.2 | 2.5 | 1.2 |
| Menopause status | | | | |  |  |
| Pre-menopausal | 0 (0%) | | 0 (0%) | 0 (0%) | 0 (0%) | 0 (0%) |
| Post-menopausal | 3 (100%) | | 167 (100%) | 1 (100%) | 1 (100%) | 1 (100%) |

| **Supplementary Table S2. Primers used in this study.** | |
| --- | --- |
| Reverse transcription PCR | |
| YAP | F: 5’-ATGGATCCCGGGCAGCAGCCGCCGCC-3’ |
|  | R: 5’-CTATAACCATGTAAGAAAGCTTTCTT-3’ |
| Real-Time Quantitative Reverse Transcription PCR | |
| CD133 | F: 5’-AGTCGGAAACTGGCAGATAGC-3 |
|  | R: 5’-GGTAGTGTTGTACTGGGCCAAT-3 |
| CCN2 | F: 5’-ACCTCTCACCGCCCTTTATCT-3' |
|  | R: 5'-GTGAAGGCGATCTTGTTGATCT-3' |
|  | R: 5’-TGTTTCTCGCTTTTCCACTGTT-3’ |
| IGFBP3 | F: 5’-AGAGCACAGATACCCAGAACT-3' |
|  | R: 5’-GGTGATTCAGTGTGTCTTCCATT-3’ |
| TGFB2 | F: 5’-CAGCACACTCGATATGGACCA -3' |
|  | R: 5’-CCTCGGGCTCAGGATAGTCT-3’ |
| FOXF2 | F: 5’-AATGCCACTCGCCCTACAC-3' |
|  | R: 5’-CGTTCTGGTGCAAGTAGCTCT-3’ |
| MYOF | F: 5’-TAATTGGCACGGCGACTGTAG-3' |
|  | R: 5’-GGAGATCAGCTTGTACGGCAG-3’ |
| CCD80 | F: 5’-GACCCCGTTTCACTATGCTGT-3' |
|  | R: 5’-GGCGAGCTAGTCTCAACACG-3’ |
| BIRC2 | F: 5’-AGCACGATCTTGTCAGATTGG-3' |
|  | R: 5’-GGCGGGGAAAGTTGAATATGTA-3’ |
| BIRC5 | F: 5’-AGGACCACCGCATCTCTACAT-3' |
|  | R: 5’-AAGTCTGGCTCGTTCTCAGTG-3’ |
| GLI2 | F: 5’-CTGCCTCCGAGAAGCAAGAAG-3' |
|  | R: 5’-GCATGGAATGGTGGCAAGAG-3’ |
| AREG | F: 5’-GTGGTGCTGTCGCTCTTGATA-3' |
|  | R: 5’-CCCCAGAAAATGGTTCACGCT-3’ |
| YWHAB promoter | F: 5’-CTGGACCAAAATAATATAAAGGCCTT-3’ |
|  | R: 5’-TCGGGGCCTAGGAAACGTGTGTG-3’ |
| YWHAB mRNA | F: 5’-ATAAAAGTGAGCTGGTACAGAAAGC-3’ |
|  | R: 5’-ATGCTGGAGATGACACGCCAGGAAGA-3’ |
| GAPDH | F: 5’-GGAGCGAGATCCCTCCAAAAT-3’ |
|  | R: 5’-GGCTGTTGTCATACTTCTCATGG-3’ |

| **Supplementary Table S3. shRNAs used in this study.** | |
| --- | --- |
| Gene | shRNA targeting sequence |
| YWHAB#1 | 5'-GGCTGAGCGATATGATGATAT-3' |
| YWHAB#2 | 5'-TGCAGCCTACACACCCAATTC-3' |
| Control | 5’-TTCTCCGAACGTGTCACGT-3’ |
